# Does Dental Fear in Children Predict Untreated Dental Caries? An Analytical Cross-Sectional Study

**DOI:** 10.3390/children8050382

**Published:** 2021-05-12

**Authors:** Suman Panda, Mir Faeq Ali Quadri, Imtinan H. Hadi, Rafaa M. Jably, Aisha M. Hamzi, Mohammed A. Jafer

**Affiliations:** 1 Division of Pediatric Dentistry, Department of Preventive Dental Sciences, Jazan University, Jazan 45142, Saudi Arabia; sumanpanda73@gmail.com; 2 Division of Dental Public Health, Department of Preventive Dental Sciences, Jazan University, Jazan 45142, Saudi Arabia; dr.mjafer@gmail.com; 3 Interns, College of Dentistry, Jazan University, Jazan 45142, Saudi Arabia; imtinan0039@gmail.com (I.H.H.); rjably11866@gmail.com (R.M.J.); aishamh95@hotmail.co (A.M.H.)

**Keywords:** dental fear, dental phobia, dental anxiety, children, oral health, dental caries, Saudi Arabia

## Abstract

Despite free health care services in Saudi Arabia, the prevalence of caries in children is substantially greater in comparison to other high-income countries. Dental fear in children may be an important issue that needs attention. Therefore, the aim was to investigate the role of dental fear in predicting untreated dental caries in schoolchildren. This analytical cross-sectional study included children aged 8–10 years residing in Saudi Arabia. Dental status via oral examinations was surveyed with the WHO standardized chart and the Children Fear Survey Schedule—Dental Subscale was used to score dental fear. Descriptive, binary, and multivariable logistic regression analyses were performed to report the findings at 5% statistical significance. Overall, there were 798 schoolchildren with an average fear score of 36. Nearly 70.4% reported fear of someone examining their mouth. About 76.9% had at least one carious tooth in their oral cavity. Children with dental fear were 1.8 times (OR = 1.80; 95% CI = 1.26, 2.56) more likely to have at least one untreated carious tooth in their oral cavity than those who did not express fear during oral examinations and dental procedures. Thus, the current study concludes that fear of dentists and dental treatment procedures successfully predicts untreated carious teeth in schoolchildren.

## 1. Introduction

The Global Burden of Disease study estimated a loss of 74,000 disability-adjusted life years (DALYs) due to dental anxiety [1]. In children, fear of dentists and dental treatments is significantly associated with delay in oral health care, most often due to increased frequency of appointment cancellations [2,3]. Though there is a noticeable difference between the terms dental fear (DF), dental anxiety (DA), and dental phobia (DP) [4], the usual fear of dentists and dental treatments in children is an important issue that needs attention. A report aggregated findings from 50 studies and estimated that nearly 23.9% of children fear dentists or dental treatments [4]. More preschoolers (36.5%) had experienced dental fear, followed by schoolchildren (25.8%) and adolescents (13.3%) [4]. If unaddressed, there could be further increases in the burden of oral health problems in children due to delayed dental visits, which may in turn negatively impact their oral health-related quality of life (OHRQoL) and wellbeing [5]. 

Saudi Arabia is a high-income nation, and the residents are entitled to free general and oral health care services. Despite this, the prevalence of oral diseases, especially dental caries in children, is substantially greater in comparison to other high-income countries [6]. It is stated that nearly 80% of children in Saudi Arabia have experienced oral health problems including dental caries [7]. Another study carried out in the capital city of Saudi Arabia showed that about 83% of children have at least one untreated carious tooth [6]. Furthermore, the prevalence of tooth decay and gum diseases was nearly the same in children of the less developed province of Jazan [8]. Thus, it is plausible that there is underutilization of free oral health services in Saudi Arabia, leading to increased oral health problems in children, and dental fear could be one of the important factors. Studies reveal that the fear of dentists and dental treatments in Saudi Arabian children is common, and is reported more in girls than boys [9]. Two recent systematic reviews state that the recent cross-sectional studies investigating the association between dental fear and oral health status in children did not recruit the participants randomly, did not adjust for oral hygiene practices, and the confidence intervals of the findings were wider, making it difficult to draw conclusions [4,10]. Additionally, there is no study that has explored the association between dental fear and caries status of schoolchildren in Saudi Arabia. Thus, the aim of this study is to investigate the role of dental fear in predicting the untreated dental caries in schoolchildren of Saudi Arabia. It is hypothesized that children who report dental fear are more likely to have untreated carious teeth in comparison to those who do not report dental fear.

## 2. Material and Method

### 2.1. Study Design and Study Setting

The current study is cross-sectional in design. Six public schools in Jazan Province of Saudi Arabia were visited to collect the data. Jazan is located at the southern part of the Arabian peninsula bordering Yemen. Questionnaire administration and oral examinations were the methods used to collect the data, and the overall data collection period lasted for two months (January to February 2020). 

### 2.2. Sample Size Calculation and Sampling Technique

Sample size calculation was based on the following parameters derived from a previous publication [9]. With a design effect of 1, precision of 0.05, and the prevalence of dental fear being 20% among children in the city of Jeddah of Saudi Arabia [9], the minimum sample size required to reject the null hypothesis was estimated at 236. As the schools in Saudi Arabia are segregated by sex and more girls in earlier studies reported to have experienced dental fear than boys, it is assumed that gender may have an effect on the findings of this study. Therefore, the current study planned to use the calculated sample size individually for each sex (N _boys_ = N girls = 236). 

A multistage stratified random sampling technique was performed. In the first stage, 4 out of the fourteen districts in Jazan Province were selected. The list of schools in the selected districts was obtained, and 4 boys’ and 4 girls’ schools were randomly selected. All children aged 8–10 years in the selected schools were invited to participate in the study, and then subjected to the selection criteria. Randomization at each step was carried out using a lottery technique. 

### 2.3. Selection Criteria

Only Saudi Arabian children aged 8–10 years were included in the study. Selection of Saudi Arabian children and a small age range contributed to the homogeneity of the sample. Further, the age group was selected to report prevalence of dental caries in mixed dentition. The children who reported existing medical conditions and those who did not provide the signed consent were excluded.

### 2.4. Data Collection Process and Variables Assessed

Dental status was reported using oral examinations and the data were surveyed with the standardized chart provided online by the World Health Organization (WHO-5th Edition) [11]. Caries experience was obtained using cumulated values of the decayed and filled teeth (D/dft index), and untreated caries were reported by adding the decayed primary (dt) and permanent teeth (DT). Three pre-calibrated dental interns (ICC_mean_ = 0.94) who were part of this study performed the oral examinations on children on the school premises. One classroom in each school was arranged for oral examinations and children with signed consent were examined under LED light using disposable mouth mirrors and probes. The interns took turns to examine the children, and those who were not performing the oral examinations assisted in data recording and questionnaire administration.

The questionnaire was divided into three sections. The first section comprised items to report the socio-demographic characteristics of the study sample (age, sex, parent’s education, parent’s work status, and family income). The second section comprised items to report the oral hygiene habits of children, and the final section comprised the Children Fear Survey Schedule—Dental Subscale (CFSS-DS) to score the dental fear of children. This scale was selected due to the ease of administration and also because its Arabic version was subjected to psychometric analyses [12]. Dental fear was assessed using 15 questions broadly concerning “dentists”, “injections”, and “opening the mouth”. Responses were coded on a 5-point Likert scale (1 = not afraid at all, 5 = very afraid). Possible scores ranged from 15 to 75; higher scores indicated greater dental fear. For more expressive interpretation of findings, the mean for each item was determined and the scores were dichotomized into no fear (below mean) vs. has fear (above mean). A second set of coded questionnaires examining the parents’ past dental experience, their educational status, and family income were given to the children to pass it on to their parents, and the filled questionnaires were collected on the following day.

### 2.5. Statistical Analysis

The data were entered and subsequently analyzed using SPSS version 24 (IBM, New York, USA). Descriptive statistics were reported as mean and standard deviation or percentages, as necessary. Binary logistic regression was carried out to investigate the association between various independent variables (sociodemographic characteristics, oral hygiene habits, and dental fear score) and dental caries status (untreated dental caries and caries experience). This was followed by multivariable logistic regression analyses to report the adjusted odds ratio values of the association between dental fear and the two outcome variables, i.e., untreated dental caries and caries experience. The statistical significance was set at five percent for all the analyses.

### 2.6. Ethical Considerations

The current study was carried out in accordance with the Declaration of Helsinki 1964, and was approved by the Ethical Review Board at Jazan University (Reference Number: REC41/1-016). Permissions to carry out oral examinations and distribution of questionnaires on the school premises were approved by the Department of Education at Jazan. Finally, all participants and their parents submitted their signed consent to be part of the study.

## 3. Results

The study sample included 798 schoolchildren, of which the majority of them were girls (70.2%), and this was because most of the boys either did not provide the signed consent or did not bring the completed questionnaire from their parents. The mean age was 8.79 years (SD = 0.79), and there were more 8-year-olds (44.4%), than 9- and 10-year-olds. A greater proportion of parents were educated to a graduate level, and only a few of them were uneducated (fathers = 4.3%; and mothers = 6.5%). Parents of 83.5% and 81.2% of children reported that their children were always in a good health and also had good oral health, respectively. However, the past dental experience of a majority of parents (75.4%) was poor (Table 1). The descriptive data on oral hygiene habits are presented in Table 1. It was observed that a large proportion of children reported brushing their teeth with fluoridated toothpaste (83.7%) and about half of them brushed their teeth twice daily (54.6%). Visiting the dentist at least once every year and never visiting the dentist were reported by 31.8% and 37.3% of the study sample, respectively.

Table 2 describes the dental fear score among the study sample. Nearly 70.4% of the children reported to have fear of someone examining their mouth and fear of someone looking at them. About 61.2% and 46.9% of them had fear of injections and fear of a dentist drilling their teeth, respectively. 

Table 3 presents the dental status of children as examined clinically. Due to mixed dentition and the selected age group, the mean value for the decayed primary teeth (3.51 ± 3.26) was estimated to be higher than the permanent teeth (1.45 ± 1.95). Similarly, the mean value for the filled primary teeth (0.48 ± 1.57) was higher than the permanent teeth (0.13 ± 0.64). There was no statistically significant difference between clinical dental parameters and parents’ perceived oral health status of children (Table 3).

For further analyses, the cumulative score of the number of decayed primary and permanent teeth was dichotomized to report the proportion of children having at least one untreated carious tooth (76.9%) vs. sound teeth (23.1%). Similarly, the cumulative scores of decayed and filled primary and permanent teeth were dichotomized to report the proportion of children having experienced caries in at least one tooth (83.0%) vs. sound teeth (17%). The binary and multivariable logistic regression analyses models were computed with untreated caries and caries experience as dependent variables. Table 4 demonstrates the association between sociodemographic characteristics, oral hygiene habits, and dental fear with the two dependent variables. It was observed that children whose parents had good past experience with dentists were less likely to have untreated carious teeth (OR = 0.65; 95% CI = 0.43, 0.97) and less likely to experience caries (OR = 0.56; 95% CI = 0.36, 0.87). An increase in the frequency of brushing was also protective of untreated carious teeth (OR = 0.59; 95% CI = 0.42, 0.82) and caries experience (OR = 0.55; 95% CI = 0.38, 0.79). Finally, children with dental fear (scoring above the mean value) had nearly a two times greater chance of having untreated carious teeth (OR = 1.85; 95% CI = 1.31, 2.60) and 1.6 times greater chance of experiencing caries (OR = 1.57; 95% CI = 1.07, 2.29).

Table 5 presents the findings from the multivariable analyses. Here, the independent variable of choice, i.e., dental fear, is positioned in the first block of the model followed by the other significant variables from the binary logistic regression analysis. The results showed that dental fear significantly predicted untreated carious teeth and caries experience after adjusting for other variables. It was observed that children with dental fear were 1.8 times (OR_adjusted_ = 1.80; 95% CI = 1.26, 2.56) and 1.5 times (OR_adjusted_ = 1.50; 95% CI = 11.01, 2.21) more likely to have at least one untreated carious tooth in their oral cavity and to have had experienced caries, respectively, than those children with no fear.

## 4. Discussion

The oral health of children is a public health concern, and poor oral health status in children will increase the burden during their life course, eventually hampering their quality of life and wellbeing [13]. Dental caries is the most common oral health problem affecting children worldwide [13]. Saudi Arabia is no different, and the prevalence is high despite the growing number of private and public dental services in the country [14,15,16,17,18]. The current analytical cross-sectional study demonstrated that the fear of dentists and dental treatments significantly predicted the presence of untreated dental caries in children. It is observed that children who reported fear of dentists and dental treatments were nearly two times more likely to have at least one untreated carious tooth in comparison to those with no fear. One explanation could be that, because of fear, children are reluctant to go to the dentist, thereby increasing the likelihood of suffering from dental caries. During the analysis, the models were adjusted for other significant confounding variables, and the precision of the findings was better than the earlier studies [4,10]. Moreover, very few of the earlier reports had explored dental fear as a predictor for dental caries. Henceforth, the findings of the current study imply a paradigm shift, and warrant further investigation of this relationship using pathway analysis. It is plausible that children whose parents had previous bad experience during a dental procedure or who do not possess adequate dental literacy may contribute to dental fear among their children [19]. This may lead to less frequent dental visits and therefore increase the burden of untreated dental caries in children. This concept best explains the situation of underutilization of free dental services by the children. In accordance with the findings of the current study, Raadal and colleagues (2002) stated that children aged 10 years, who fear dentists or dental treatments, were more likely to develop caries [20]. Another study presented a significant association between the number of filled teeth and dental fear in children aged 3–14 years [21]. Accordingly, one finding of the present study indicated that past dental experience of parents was significantly associated with the dental status of children. Therefore, it is essential that parents and children are made familiar with the role of dentists and dental procedures, as this may possibly reduce their anxiety, and subsequently increase the visits to dentists for preventive and treatment services.

The mean fear score (CFSS-DS) in the current report was in agreement to previous studies (mean = 25.99 ± 9.31) [9,22]. Additionally, the cut-off value (mean = 36) to describe presence or absence of fear in the current study sample is similar to studies that were performed in Sweden (mean ≥ 38) [23]. However, the fear score is greater than in Dutch children [24], and lower in comparison to children in the United States [25] and Japan [26]. Importantly, it can be seen that nearly 60% of the children in the current study were fearful of injections, and this resembles the findings from the earlier studies [27,28]. Furthermore, severe phobia is reported in around 5–20% of children from other countries [25,29]. In one qualitative study, a girl quotes ‘*I completely have phobia of needle*’ [30]. Hence, knowing that dental fear acquired in childhood may continue in adulthood, it is important to address the issue at the earliest point. Moreover, children with phobia may be uncooperative during their visit to dental clinics, creating occupational stress for dental professionals.

The findings of the current study are exclusive, and should be interpreted within the context of its methodological strengths and limitations. The current study is the first from Saudi Arabia to establish a relationship between dental fear and dental caries. The data were collected from a large sample of a homogenized population, and validated tools were used in assessing dental fear and also to report dental status. The multistage random sampling technique contributed to the validity of the findings. Further, the association was adjusted for sociodemographic characteristics and oral hygiene habits. Nonetheless, it is a cross-sectional study, and a causal association between dental fear and dental caries cannot be established. Additionally, dental caries is a multifactorial disease and dental fear or anxiety may not be a standalone factor for the occurrence of the disease. Lastly, the findings may not be extrapolated to the study samples from other nations, especially developed nations where children visit dentists more regularly.

To conclude, the findings of the current study show that dental fear in children predicts their dental status. Children who fear dentists and dental treatments are more likely to suffer from untreated carious teeth. As dental caries is multifactorial, the mechanism that explains the link between dental fear and dental caries needs to be further explored. It is plausible that less frequent dental visits, poor dietary habits, and poor oral hygiene behavior mediate the link between dental fear and dental caries. Additionally, it is important to consider the dental literacy of parents, their past dental experience, level of education, and childrearing as they may be contributing factors in the relationship.

Therefore, it is suggested that community-based programs be implemented to familiarize children and their parents with dental procedures. Preparing them psychologically for dental procedures should be the starting point, as it may possibly reduce their level of anxiety during dental visits and contribute towards a positive dental experience. Evidence-based cognitive behavioral techniques can be a guided therapy to address dental fear and anxiety among children. Though, dental practitioners should be more sensitive in their patient approach, especially while treating children, it is important that underlying factors like improved parenting, dental literacy, dietary behaviors, and oral hygiene practices should also be addressed using effective public health programs.

This study evaluates the association between dental fear in children and their dental status. Based on the findings, it is understood that fear of dentists and dental treatment procedures in children is one of the key predictors of untreated carious teeth.

It is also observed that children whose parents have had a previous good dental experience are less likely to have untreated carious teeth.

All dentists, especially pediatric dental specialists, should continue to actively address dental fear of parents and children who visit their dental practices.

Effective public health programs are required in addressing underlying factors like improved parenting, dental literacy dietary behaviors, and oral hygiene practices, as these are the contributing factors in the relationship between dental fear and dental caries.

## Figures and Tables

**Table 1:** Sociodemographic characteristics and oral hygiene habits of the study sample (N = 798).

Sociodemographic Characteristics and Oral Hygiene Habits	N (%)
**Sex**	
Boys	238 (29.8%)
Girls	560 (70.2%)
**Age**	
8-year-olds	354 (44.4%)
9-year-olds	260 (32.6%)
10-year-olds	184 (23.1%)
**Father’s Level of Education**	
Post-graduate	288 (36.1%)
Graduate	344 (43.1%)
Schooling	132 (16.5%)
Uneducated	34 (4.3%)
**Mother’s Level of Education**	
Post-graduate	262 (32.8%)
Graduate	260 (32.6%)
Schooling	224 (28.1%)
Uneducated	52 (6.5%)
**Family Income Per Month**	
Equal or more than SAR 10,000	344 (43.1%)
Less than SAR 10,000	452 (56.6%)
No formal income	2 (0.3%)
**Parent’s Perception of Children’s General Health**	
Always in good health	666 (83.5%)
Is unhealthy sometimes	132 (16.5%)
**Parent’s Perception of Children’s Oral Health**	
Good	648 (81.2%)
Poor	150 (18.8%)
**Past Dental Experience of Parents**	
Good	196 (24.6%)
Poor	602 (75.4%)
**Mode of Teeth Cleaning**	
Toothbrush with fluoridated paste and dental floss	74 (9.3%)
Toothbrush with fluoridated paste	668 (83.7%)
None *	56 (7.0%)
**Frequency of Brushing (Per Day)**	
Two times or more	436 (54.6%)
Once daily	274 (34.3%)
Zero times *	88 (11.0%)
**Amount of Fluoridated Toothpaste Used**	
Covers the full toothbrush	362 (45.4%)
Covers half the toothbrush	302 (37.8%)
Size of a pea	96 (12.0%)
Less than the size of a pea	38 (4.8%)
**Duration of Brushing**	
Between 30 s to one minute	446 (55.9%)
Between one to two minutes	164 (20.6%)
More than two minutes	92 (11.5%)
Do not know	96 (12.0%)
**Frequency of Dental Visits**	
Twice a year	32 (4.0%)
One time a year	254 (31.8%)
When there is toothache	214 (26.8%)
Never	298 (37.3%)

* Comprises no tooth brushing, no flossing, and/or using only miswak.

**Table 2:** Dental fear among children assessed using the CFSS-DS tool (N = 798).

CFSS-DS Item	No Fear (Below Mean)	Has Fear (Above Mean)
Fear of dentists	530 (66.4%)	268 (33.6%)
Fear of doctors	522 (65.4%)	276 (34.6%)
Fear of injections	310 (38.8%)	488 (61.2%)
Fear of having somebody examine your mouth	236 (29.6%)	562 (70.4%)
Fear of having to open your mouth	520 (65.2%)	278 (34.8%)
Fear of having a stranger touch you	536 (67.2%)	262 (32.8%)
Fear of having somebody look at you	236 (29.6%)	562 (70.4%)
Fear of dentist drilling	424 (53.1%)	374 (46.9%)
Fear of the sight of dentist drilling	424 (53.1%)	374 (46.9%)
Fear of the noise of dentist drilling	464 (58.1%)	334 (41.9%)
Fear of having somebody put instruments in your mouth	426 (53.4%)	372 (46.6%)
Fear of choking	478 (59.9%)	320 (40.1%)
Fear of having to go to the hospital	444 (55.6%)	354 (44.4%)
Fear of people in white uniforms	528 (66.2%)	270 (33.9%)
Fear of having the nurse clean your teeth	500 (62.7%)	298 (37.3%)

**Table 3:** Relationship between clinical dental parameters and parents’ perceived oral health status of children.

Dental Parameters (dft)	Mean (S.D)	Parents’ Perceived Oral Health Status			
		*p*-Value	OR	95% CI	
				Lower	Upper
Decayed permanent teeth	1.45 (1.95)	0.11	0.66	0.39	1.11
Decayed primary teeth	3.51 (3.26)	0.37	1.27	0.75	2.13
Filled permanent teeth	0.13 (0.64)	0.29	1.76	0.62	4.98
Filled primary teeth	0.48 (1.57)	0.24	1.39	0.80	2.41
Sound permanent teeth	10.21 (2.37)	0.57	0.86	0.51	1.44
Sound primary teeth	7.75 (3.38)	0.25	1.36	0.81	2.29

dft: decay and filled teeth; OR: Odds ratio; 95% CI: Confidence interval (precision).

**Table 4:** Binary logistic regression models demonstrating the association between dental caries status and dental caries experience with various independent variables.

Independent Variable	Untreated Caries			Caries Experience		
	Odds Ratio	95% CI	*p*-Value	Odds Ratio	95% CI	*p*-Value
Gender (boys)	0.75	0.50, 1.14	0.18	1.27	0.82, 1.97	0.28
Age	0.88	0.70, 1.10	0.27	0.92	0.72, 1.18	0.52
Father’s Level of Education	0.91	0.69, 1.20	0.50	1.07	0.79, 1.45	0.66
Mother’s Level of Education	0.90	0.71, 1.14	0.37	0.87	0.67, 1.13	0.29
Family Income Per Month	0.69	0.46, 1.03	0.07	0.74	0.48, 1.15	0.18
Past Dental Experience of Parents (good)	0.65	0.43, 0.97	0.04	0.56	0.36, 0.87	0.011
Mode of Teeth Cleaning						
Toothbrush with fluoridated paste and dental floss	19.15	0.71, 98.78	0.18	3.02	0.37, 78.25	0.18
Toothbrush with fluoridated paste	2.03	0.13, 3.63	0.27	1.42	0.60, 4.74	0.11
None *	1					
Frequency of Brushing (per day)	0.59	0.42, 0.82	0.002	0.55	0.38, 0.79	0.001
Amount of Fluoridated Toothpaste Used	1.72	0.38, 2.15	0.19	1.41	0.11, 1.78	0.15
Duration of Brushing	1.59	0.24, 2.04	0.20	1.71	0.28, 2.27	0.18
Frequency of Dental Visit	0.87	0.72, 1.05	0.15	1.10	0.89, 1.37	0.36
Having Dental Fear (yes)	1.85	1.31, 2.60	<0.001	1.57	1.07, 2.29	0.02

* Comprises no tooth brushing, no flossing, and/or using only miswak; 95% CI: Confidence interval (precision).

**Table 5:** Multivariable logistic regression models demonstrating the association between dental caries status and dental caries experience with various independent variables.

Independent Variable	Untreated Caries			Caries Experience		
	Adjusted Odds Ratio	95% CI	*p*-Value	Adjusted Odds Ratio	95% CI	*p*-Value
Having Dental Fear (yes)	1.80	1.26, 2.56	0.001	1.50	1.01, 2.21	0.04
Past Dental Experience of Parents (good)	0.65	0.44, 0.98	0.04	0.58	0.38, 0.89	0.01
Frequency of Brushing (per day)	0.56	0.41, 0.77	<0.001	0.56	0.39, 0.79	0.001

95% CI: Confidence interval (precision).

## Data Availability

The data is available upon reasonable request.

## References

[1] Vermaire J.H., van Houtem C.M.H.H., Ross J.N., Schuller A.A. (2016). The burden of disease of dental anxiety: Generic and disease-specific quality of life in patients with and without extreme levels of dental anxiety. Eur. J. Oral. Sci..

[2] Armfield J.M., Heaton L.J. (2013). Management of fear and anxiety in the dental clinic: A review. Aust. Dent. J..

[3] Ten Berge M. (2008). Dental fear in children: Clinical consequences. Suggested behaviour management strategies in treating children with dental fear. Eur. Arch. Paediatr. Dent..

[4] Grisolia B.M., dos Santos A.P.P., Dhyppolito I.M., Buchanan H., Hill K., Oliveira B.H. (2020). Prevalence of dental anxiety in children and adolescents globally: A systematic review with meta-analyses. Int. J. Paediatr. Dent..

[5] Merdad L., El-Housseiny A.A. (2017). Do children’s previous dental experience and fear affect their perceived oral health-related quality of life (OHRQoL)?. BMC Oral Health.

[6] Alhabdan Y.A., Albeshr A.G., Yenugadhati N., Jradi H. (2018). Prevalence of dental caries and associated factors among primary school children: A population-based cross-sectional study in Riyadh, Saudi Arabia. Environ. Health Prev. Med..

[7] Al Agili D.E. (2013). A systematic review of population-based dental caries studies among children in Saudi Arabia. Saudi Dent. J..

[8] Bokhari A.M., Quadri M.F.A. (2020). What factors contribute to the self-reported oral health status of Arab adolescents? An assessment using a validated Arabic-WHO tool for child oral health (A-OHAT). BMC Oral Health.

[9] Alshoraim M.A., El-Housseiny A.A., Farsi N.M., Felemban O.M., Alamoudi N.M., Alandejani A.A. (2018). Effects of child characteristics and dental history on dental fear: Cross-sectional study. BMC Oral Health.

[10] Cianetti S., Lombardo G., Lupatelli E., Pagano S., Abraha I., Montedori A., Caruso S., Gatto R., Giorgio S.D., Salvato R. (2017). Dental fear/anxiety among children and adolescents. A systematic review. Eur. J. Paediatr. Dent..

[11] WHO, World Health Organization (2013). Oral Health Surveys-Basic Methods.

[12] Alharbi A., Humphris G., Freeman R. (2019). The psychometric properties of the CFSS-DS for schoolchildren in Saudi Arabia: A confirmatory factor analytic approach. Int. J. Paediatr. Dent..

[13] Peres M.A., Macpherson L.M.D., Weyant P.R.J., Daly P.B., MSc R.V., Mathur M.R., Listl S., Celeste R.K., Guarnizo-Herreño C.C., Kearns C. (2019). Oral diseases: A global public health challenge. Lancet.

[14] World Health Organization, Regional Office for the Eastern Mediterranean (2009). Towards a Strategy for Cancer Control in the Eastern Mediterranean Region.

[15] Quadri F., Hendriyani H., Pramono A., Jafer M. (2015). Knowledge, attitudes and practices of sweet food and beverage consumption and its association with dental caries among schoolchildren in Jazan, Saudi Arabia. East Mediterr. Health J..

[16] Quadri F.A., Jafari F.A., Albeshri A.T., Zailai A.M. (2018). Factors influencing Patients’ Utilization of Dental Health Services in Jazan, Kingdom of Saudi Arabia. Int. J. Clin. Pediatr. Dent..

[17] Quadri M.F.A., Shubayr M.A., Hattan A.H., Wafi S.A., Jafer A.H. (2018). Oral Hygiene Practices among Saudi Arabian Children and Its Relation to Their Dental Caries Status. Int. J. Dent..

[18] Zailai A.M., Quadri M.F.A., Nayeem M., Inamdar A., Tadakamadla S. (2014). Caries status of school children in Jazan city, KSA and its relation with dental literacy of their parents. J. Oral Health Res..

[19] Tadakamadla S.K., Quadri M.F.A., Pakpour A.H., Zailai A.M., Sayed M.E., Inamdar M.M.A.S., Tadakamadla J. (2014). Reliability and validity of Arabic Rapid Estimate of Adult Literacy in Dentistry (AREALD-30) in Saudi Arabia. BMC Oral Health.

[20] Raadal M., Strand G.V., Amarante E.C., Kvale G. (2002). Relationship between caries prevalence at 5 years of age and dental anxiety at 10. Eur. J. Paediatr. Dent..

[21] Khawja S.G., Arora R., Shah A.H., Wyne A.H., Sharma A. (2015). Maternal Dental Anxiety and its Effect on Caries Experience Among Children in Udaipur, India. J. Clin. Diagn. Res..

[22] El-Housseiny A.A., Alsadat F.A., Alamoudi N.M., Derwi D.A.E., Farsi N.M., Attar M.H., Andijani B.M. (2016). Reliability and validity of the Children’s Fear Survey Schedule-Dental Subscale for Arabic-speaking children: A cross-sectional study. BMC Oral Health.

[23] Dahlander A., Soares F., Grindefjord M., Dahllöf G. (2019). Factors Associated with Dental Fear and Anxiety in Children Aged 7 to 9 Years. Dent. J..

[24] Ten Berge M., Veerkamp J.S., Hoogstraten J. (2002). The etiology of childhood dental fear: The role of dental and conditioning experiences. J. Anxiety Disord..

[25] Klingberg G. (1995). Dental fear and behavior management problems in children. A study of measurement, prevalence, concomitant factors, and clinical effects. Swed. Dent. J. Suppl..

[26] Nakai Y., Hirakawa T., Milgrom P., Coolidge T., Heima M., Mori Y., Ishihara C., Yakushiji N., Toshiko Yoshida T., Shimono T. (2005). The Children’s Fear Survey Schedule-Dental Subscale in Japan. Community Dent. Oral Epidemiol..

[27] Humphris G.M., Morrison T., Lindsay S.J. (1995). The Modified Dental Anxiety Scale: Validation and United Kingdom norms. Community Dent. Health.

[28] Wogelius P., Rosthøj S., Dahllöf G., Poulsen S. (2009). Dental anxiety among survivors of childhood cancer: A cross-sectional study. Int. J. Paediatr. Dent..

[29] Lee C.Y., Chang Y.Y., Huang S.T. (2007). Prevalence of dental anxiety among 5- to 8-year-old Taiwanese children. J. Public Health Dent..

[30] Gao X., Hamzah S., Yiu C.K.Y., McGrath C., King N.M. (2013). Dental fear and anxiety in children and adolescents: Qualitative study using YouTube. J. Med. Internet Res..

